# Strategies to reach and motivate migrant communities at high risk for TB to participate in a latent tuberculosis infection screening program: a community-engaged, mixed methods study among Eritreans

**DOI:** 10.1186/s12889-020-8390-9

**Published:** 2020-03-12

**Authors:** Ineke Spruijt, Dawit Tesfay Haile, Connie Erkens, Susan van den Hof, Simone Goosen, Andrea ten Kate, Hewan Teshome, Marja Karels, Marga Koenders, Jeanine Suurmond

**Affiliations:** 1 KNCV Tuberculosis Foundation, The Hague, The Netherlands; 2 Department of Public Health, Amsterdam Public Health Research Institute, Amsterdam University Medical Centres, University of Amsterdam, Amsterdam, The Netherlands; 3 Present Address: National Institute for Public Health and the Environment (RIVM), Centre for Infectious Disease Control, Bilthoven, The Netherlands; 4 Netherlands Association of Community Health Services, Utrecht, The Netherlands; 5 Department of Tuberculosis Control, Public Health Service IJsselland, Zwolle, The Netherlands; 6 Department of Tuberculosis Control, Public Health Service Haaglanden, Den Haag, The Netherlands; 7 Department of Tuberculosis Control, Public Health Service Hollands Noorden, Alkmaar, The Netherlands; 8 Department of Tuberculosis Control, Public Health Service Gelderland Zuid, Nijmegen, The Netherlands

**Keywords:** Tuberculosis, Latent tuberculosis infection, LTBI, Screening, Eritreans, Migrants, Community-engaged, Refugees

## Abstract

**Background:**

In the Netherlands, migrant populations with a high tuberculosis (TB) incidence are an important target group for TB prevention. However, there is a lack of insight in effective community-engaged strategies to reach and motivate these migrants to participate in latent TB infection (LTBI) screening and treatment programs.

**Methods:**

In cocreation with Eritrean key figures and TB staff, we designed and executed six strategies to reach and motivate Eritrean communities to participate in LTBI programs, in five regions in the Netherlands. We registered participation in LTBI education and screening, and LTBI treatment uptake and completion. We used semi-structured group and individual interviews with Eritrean participants, key figures, and TB staff to identify facilitators and barriers.

**Results:**

Uptake of LTBI education (13–75%) and consequent screening (10–124%) varied between strategies. LTBI screening uptake > 100% resulted from educated participants motivating others to participate in screening. Two strategies, using face-to-face promotion and targeting smaller groups, were the most successful. The program resulted in high LTBI treatment initiation and completion (both 97%). Reported program barriers included: competing priorities in the target group, perceived good health, poor risk perception, and scepticism towards the program purpose. TB staff perceived the program as useful but demanding in terms of human resources.

**Conclusions:**

Eritrean migrant communities can be successfully reached and motivated for LTBI screening and treatment programs, when sufficient (human) resources are in place and community members, well-connected to and trusted by the community, are engaged in the design and execution of the program.

## Background

In most low tuberculosis (TB) incidence countries, the majority (74% in the Netherlands) of TB is foreign born [1] and TB rates in this group remain high for at least 5 to 10 years after arrival [2, 3]. Therefore, to stimulate further decreases in TB incidence, prevention of TB through screening and treatment of latent TB infection (LTBI) among high-risk migrants is suggested as a strategy for low TB incidence countries [4, 5]. In the Netherlands, LTBI screening and treatment among migrants at arrival is feasible [6, 7]. Dutch TB policy advisors are therefore considering to replace current mandatory radiological TB entry-screening among migrants by LTBI screening and treatment [4]. However, this policy change does not target the large pool of persons with LTBI among settled migrants, who account for 60% of annual TB patients [8]. Therefore, one can argue that this group of settled, high-TB-risk migrants can also be considered as a target group for TB prevention.

Reaching and motivating migrant communities to participate in TB screening programs is not without difficulty. Barriers for screening uptake include low perceived susceptibility, stigmas and misconceptions about the disease; unfamiliarity with the concept of screening and preventive care; and limited attention to language barriers and cultural sensitivities [9–11]. To reach the population in question, engaging community members or community-based organizations, which are trusted by the community, can help overcome these barriers: they have access to the community; can channel information; educate and mobilize their community; and (hereby) alleviate stigma and promote the benefits of TB screening [5, 10, 12–16]. Therefore, designing a culturally-tailored LTBI education and screening program that engages the community and other stakeholders would be a promising way to offer LTBI screening and treatment to settled migrants [10, 17].

Since 2010, a high influx of Eritrean asylum seekers — mainly young adult men, literate but with low levels of education, and of Tigrinya ethnicity — have applied for and were granted asylum. Subsequently, many family members followed and entered the Netherlands through the family reunification program [13]. Despite a moderate estimated TB incidence rate of 89/100,000 population in Eritrea [18], this cohort of Eritrean migrants have a high TB incidence in the Netherlands [1, 2]. Therefore, they are a target population for TB prevention in the Netherlands. Although various interventions among migrant populations have been described in literature, we lack insight into which specific strategies — tailored to specific communities — will best promote the uptake of LTBI education, screening and treatment. Therefore, to reach and motivate Eritreans living in Dutch communities to participate in an LTBI education, screening and treatment program, we engaged local Eritrean key figures and TB care staff to identify and carry out tailored strategies.

## Methods

### Aim, design and setting

We used a community-engaged mixed-methods study design to develop and study strategies to reach Eritrean migrants, and motivate them to participate in an LTBI education, screening and treatment program. A community-engaged research approach involves community members who actively participate in generating ideas, contribute to decision-making, and share responsibility in the design and execution of a culturally appropriate program [12, 19].

Eritrean migrants with a maximum duration of stay in the Netherlands of 10 years were eligible for this study, as we expect this group to be at highest risk for TB compared to those who migrated more than 10 years ago [1, 2, 20]. We approached Public Health Services (PHS) — which is responsible for TB care and prevention activities — with large Eritrean communities in their region (seven PHS out of 25), of whom five agreed to participate in this study.

### LTBI education screening and treatment program

TB and LTBI screening and treatment activities were performed following the Dutch guidelines [21, 22], and consisted of three components: 1) TB and LTBI education, 2) LTBI screening and 3) LTBI treatment. The education and written materials were provided in Tigrinya, the Eritrean mother tongue. LTBI screening and treatment were offered free of charge. Additional file 1 gives a detailed overview of program procedures [see Additional file 1].

#### TB and LTBI education program

Based on experiences of Eritreans described in previous studies [6, 7], authors IS and DTH designed an education program on TB and LTBI. It consisted of a presentation, a short film [23], and interactive quizzes. DTH invited Eritreans from his social network to participate in 3 group discussions (respectively with *n* = 11, *n* = 4, *n* = 3) in which we piloted and discussed the education program. Participants were mainly young adult men (only three females participated) and received a €10 voucher for their participation. According to the group discussions, we revised the education program and consequently gave a one-day training to seven Eritrean key figures (see below), hired by the project, to provide the education as part of the program. The LTBI education was organized on site.

#### LTBI screening

LTBI screening consisted of a health questionnaire, completed by participants, and QuantiFERON-TB Gold Plus (QFT-Plus; Qiagen, Germantown, MD) [22]. To allow participants to make an independent and informed decision on participation in the screening, the PHS organized the LTBI screening, on site or at the PHS, at least 2 days after the education session of the PHS.

#### LTBI treatment

The PHS invited all participants with a QFT-plus test result ≥0.35 IU/ml for consultation with the TB physician. TB physicians confirmed LTBI diagnosis after exclusion of TB disease and offered eligible participants a three-month daily Isoniazid and Rifampicin combination treatment. TB nurses provided LTBI treatment support through regular contact based on the client’s needs.

### Community engagement and strategies

We engaged seven Eritrean community members — four adult females, one young adult man and two middle aged men — who spoke both Dutch and Tigrinya. They functioned as key figures in the development and execution of strategies to reach and motivate the Eritrean community. The key figures were, as such, already under contract with the PHS for other health-related projects. The key figures were active members of this study’s PHS project teams, which further consisted of a TB nurse (who functioned as the PHS project coordinator), the primary researcher (IS), and additional PHS staff. In a first project team meeting, key figures explained the characteristics of the Eritrean community within the PHS region, such as age, male/female ratio, and community size. Additionally, the key figures identified places and gatherings where community members would regularly come together. Based on this information, the project team discussed strategies to reach and motivate the community members, including the number of people who could potentially be reached. Next, a plan of action was designed and discussed in a second project team meeting. Table 1 provides a detailed description of the strategies used by the PHSs.

**Table 1:** Strategies to reach and motivate Eritrean migrants living in Dutch communities

Strategies	Description of strategy	PHS
***Strategy 1:****Invitation through mail and social media*	The local community of PHS 1 lacked regular social gatherings, for example a church, which could be used as approaching strategy. Therefore, the PHS 1 project team approached the target group through individual invitation -consisting of a flyer in Tigrinya- by mail, for which addresses of the target group where obtained by the PHS through the municipality. Additionally, the key figure posted an invitation on a Facebook group for Eritreans in that city (approximately 120 members). *LTBI education: organized twice, during a week night, at two different local community centres* *LTBI screening: approximately 1 week after education, on appointment, at the PHS* One key figure of PHS 3 promoted the education session in a WhatsApp group of the church. (Additional to strategy 2)	1,3
***Strategy 2:****Face-to-face promotion*	The key figure of PHS 1 asked other key figures -working for other PHS departments- to spread the invitation and promote participation within their network during face-to-face contacts. (Additional to strategy 1) The project team of PHS 3 identified various places - Dutch language classes, libraries, the church, and the gym- where Eritreans regularly gather. At those places, key figures approached individuals to promote the upcoming education session verbally and by handing out flyers with invitations. *LTBI education: organized twice, during week night, at a local community centre* *LTBI screening: organized three times, approximately 1 week after the education, during week day, at the PHS*	1,3
***Strategy 3:****Dutch language classes*	PHS 1 and 2 used Dutch language classes (PHS 1 at one school, PHS 2 at two schools) to reach Eritrean migrants. The project team approached the school management to discuss the possibility to organize education sessions at the school. After agreement, a teacher (Strategy 3.1 - PHS 1) or the key figures (Strategy 3.2 - PHS 2) approached students to come to the education session and handed out flyers. One school handed out flyers and displayed posters in the school (Strategy 3.3 - PHS 2) to promote the education session. *LTBI education: organized three times, at two different schools* *LTBI screening: approximately 1 week after the education session, once during week night at the PHS, twice on appointment during week day at the PHS*	1,2
***Strategy 4:****Group housing*	The key figures of PHS 2 (Strategy 4.1 and 4.2) and the TB nurse of PHS 4 (Strategy 4.3) utilized existing contacts with resident(s) of group housings. Group housings are temporary residents with up to 35 young adult females or males, who transferred from an asylum seeker centre and are waiting individual housing to come available. In consultation with the residents, the key figures organized an education session in a community space of the houses. *LTBI education: during a week night, at the house* *LTBI screening: organized approximately 1 week after the education, during a week day at the house (strategy 4.3 (PHS 4)) or on appointment at the PHS (strategy 4.1 and 4.2 (PHS 2))*	2,4
***Strategy 5:****Sports club*	The TB nurse of PHS 2 approached an Eritrean soccer coach who organizes weekly soccer trainings for Eritrean migrants. In consultation with the coach, the TB nurse organized an education session after soccer training. *LTBI education: during a week night, after training at the sport club* *LTBI screening: organized approximately 1 week after the education, during a week night, at the PHS*	2
***Strategy 6:****Eritrean church*	Strategy 6.1: One PHS4 key figure was a member of the church board of trustees and obtained their consent to promote the LTBI education and screening after a church service. Interested church members were asked to sign up for the screening. Registered members received an invitation by mail. Those who did not show-up for the first screening appointment were invited a second time. Strategy 6.2: The key figure of PHS 4 brought the project researcher (IS) in contact with a priest of a church in the PHS 5 region. The priest allowed the team to promote the LTBI education and screening after a church service. After the promotion, church members were handed-out invitations with date and time of screening. *LTBI education: promotion of the intervention organized after the church service* *LTBI screening: organized 1 week (PHS 4) / 2 weeks (PHS 5) after education session on appointment at the PHS* We arranged for church members who did not live in the PHS 4 or PHS 5 region to visit the PHS in their own region.	4,5

*LTBI* Latent tuberculosis infection, *PHS* Public Health Service, *TB* Tuberculosis

#### Quantitative data collection and analysis

During the screening, all participants completed health questionnaires including additional information on level of education and household composition. For each person eligible for LTBI treatment, TB physicians completed questionnaires about language barriers, occurrence of side-effects, challenges experienced during the treatment and, if applicable, reason(s) for discontinuing LTBI treatment. We double entered data from questionnaires in MS-Access (Microsoft Corp, Seattle 206 WA, USA). Additionally, we collected data from the electronic TB client registration of the PHS and from the Netherlands TB register. Before analysis, all data was merged, validated, cleaned and completely anonymized. We calculated proportions for participants’ characteristics and the cascade of care, including reasons for not initiating or completing LTBI treatment.

#### Qualitative data collection and analyses

We used semi-structured group interviews with the project teams (*n* = 4), and individual interviews (*n* = 10) plus group interviews (*n* = 5) with Eritrean participants, to evaluate strategies and identify LTBI screening and treatment program facilitators and barriers (Table 2). After familiarization with the data, we developed and refined schemes to guide the coding of transcripts from the interviews. In regular meetings authors DTH, IS and JS discussed coding, categories and interpretation of the data [24]. We used MAXQDA (Version 11, VERBI GmbH, Berlin, Germany) to assist in analyses of qualitative data. Additional file 2 provides the topic guides for the group and individual interviews [see Additional file 2].

**Table 2:** Qualitative research methods

**Group interviews with project teams (*n*** **= 4)**^**a**^	
Participation	The PHS project coordinator, the key figure(s), additional PHS staff (such as the TB physician, TB nurse, Medical Technical Assistant).
Time	Approximately 1 h
Location	At the PHS office
Informed consent	A-priori audio-taped verbal consent
Communication	Dutch
Transcript	Verbatim in Dutch (by IS)
Incentive	None
**Group interviews with Eritrean participants (*n*** **= 5)**	
Participation	Group interviews, each consisting of 4 to 6 participants, took place immediately following the LTBI screening
Time	Between 30 and 45 min
Location	At the PHS, in a separate room to ensure privacy
Informed consent	Written a-priori informed consent
Communication	Tigrinya
Transcript	Verbatim translated from Tigrinya in English (by DTH)
Incentive	None (beverages were provided)
**Individual interviews with Eritrean participants diagnosed with LTBI (*n*** **= 10)**	
Participation	TB nurses asked Eritrean clients on LTBI treatment for consent to be approached by phone for an invitation to participate in an individual interview and to set an appointment if willing to participate.
Time	Between 15 and 30 min
Location	Location to the client’s convenience
Informed consent	Written a-priori informed consent
Communication	Tigrinya
Transcript	Verbatim translated from Tigrinya in English (by DTH)
Incentive	A 10-euro voucher

*LTBI* Latent tuberculosis infection, *PHS* Public Health Service, *TB* Tuberculosis

^a^One project team (PHS 5) was not interviewed because activities were organized, in consultation with the PHS 5 TB care staff, ad-hoc by the authors IS and DTH

## Results

### Quantitative results

We estimated to reach a total of 904 Eritrean migrants through all strategies employed in the five PHS settings. In total, 401 (44%) persons attended LTBI education and 257 persons (64% of attendees, 28% of number envisioned to reach) received LTBI screening. The uptake of LTBI education differed between strategies from 13% (Strategy 3.3) to 75% (Strategy 5) (Table 3). Invitations through mail and social media (strategy 1) and church meetings (strategy 6.1 and 6.2) were most promising in reaching large numbers of the target population. However, only strategy 6.1 succeeded in screening many persons (*n* = 70). Strategies 2.1, 2.2 (face-to-face), 4.3 (group housing) and 5 (sport club) were most successful and screened respectively 84, 89, and 50% of the envisioned target group. PHS staff encouraged individuals who participated in the education sessions of strategies 2.1, 2.2, and 4.3 to motivate and bring family and friends to the LTBI screening. This resulted in more individuals attending the LTBI screening than the education session (uptakes up to 124% of those educated).

**Table 3:** Uptake of LTBI education and screening, ranked from most successful to least successful strategy

PHS	Strategies	Numbers envisioned to reach	Participated in LTBI education	Received LTBI screening	
		n	n (% of n envisioned to reach)	n (% of n LTBI education)	(% of n envisioned to reach)
**Total**		**904**	**401 (44%)**	**257 (64%)**	**(28%)**
4	**Strategy 4.3:** Female group house	35	25 (71%)	31 (124%)^a^	(89%)
3	**Strategy 2.1:** Face to face promotion	47	30 (64%)	62 (124%)^a^	(84%)
	**Strategy 2.2:** Face to face promotion	27	20 (74%)		
2	**Strategy 5:** Male football team	20	15 (75%)	10 (67%)	(50%)
2	**Strategy 4.1:** Female group house	20	12 (60%)	9 (75%)	(45%)
4	**Strategy 6.1:** Eritrean church	200	65+ (33%) ^b,c^	70 (108%)	(35%)
2	**Strategy 3.2:** Dutch language classes	50	30 (60%)	16 (53%)	(32%)
1	**Strategy 1:** Invitation through mail and social media	175	44 (25%)	32 (73%)	(18%)
1	**Strategy 3.1:** Dutch language classes	20	12 (60%)	3 (25%)	(15%)
2	**Strategy 3.3:** Dutch language classes	60	8 (13%)	7 (88%)	(12%)
2	**Strategy 4.2:** Male group house	50	30 (60%)	5 (17%)	(10%)
5	**Strategy 6.2:** Eritrean church	200	110 (55%)^d^	11 (10%)	(6%)

*PHS* Public Health Service, *LTBI* Latent tuberculosis infection

^a^Persons who attended the education session were encouraged to motivate and bring friends and family to the LTBI screening, which resulted in LTBI screening uptake (compared to LTBI education uptake) percentages over 100%

^b^Persons in the church who registered-after promotion talk after church service- to receive an invitation by mail for extensive education session and LTBI screening at the PHS

^c^One household member had to register to receive an invitation which was valid for the whole household

^d^Number of invitations handed out after the promotion talk after the church service

**Fig. 1:**
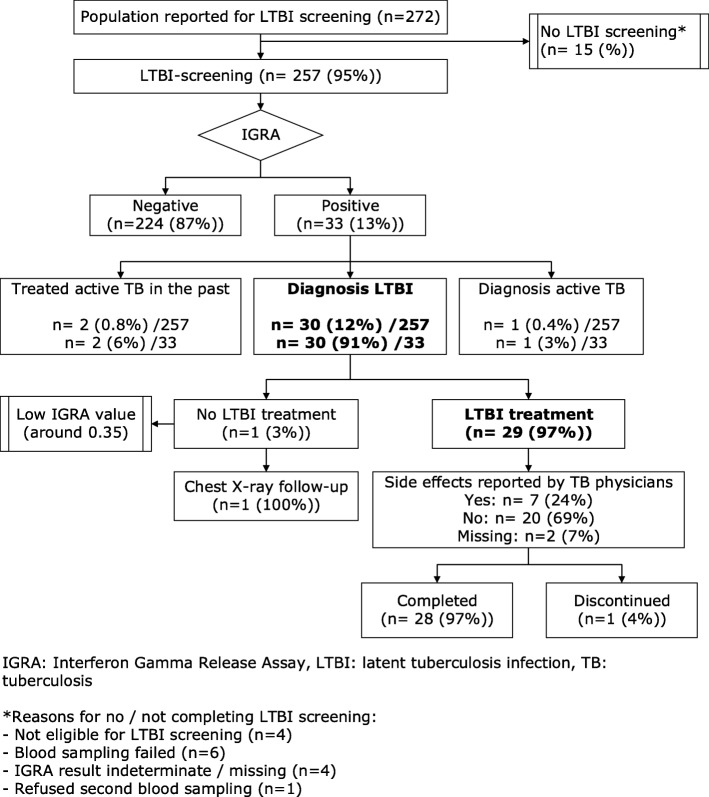
LTBI screening and treatment cascade of care

Of 257 persons screened for LTBI, 30 (12%) were diagnosed with LTBI (Fig. 1). Additional file 3 presents characteristics of the population screened and treated for LTBI [see Additional file 3]. Of those diagnosed with LTBI, 29 (97%) participants started and 28 (97%) completed LTBI treatment (Fig. 1). Seven (24%) participants had reported side-effects (hepatotoxicity: *n* = 3). To overcome language barriers, professional interpreters translated during 13 (45%) consultations, while Ethiopian/Eritrean TB nurses from the PHS translated during 14 (48%) consultations. All clients received demand-driven LTBI treatment support. Additional file 4 shows results from LTBI treatment evaluation [see Additional file 4].

### Qualitative results

#### Overall experience with the program

Overall, interview respondents appreciated the opportunity to be educated and tested for LTBI. They perceived the education as eye-opening and important, and hoped the program would continue to reach more Eritreans. Some respondents expressed their desire to be tested for other diseases, particularly HIV. Participants who received the LTBI treatment perceived the treatment support as important and respectful. Furthermore, respondents were thankful for the reimbursement of screening and treatment costs; some indicated they would not have been able to cover those costs themselves.

*Eritrean respondent: “I think it is a huge support for us to get it for free! How would we have paid for this? I don't know if these medicines exist in our country? So, I consider myself lucky to get this opportunity.” [Individual interview 1]*

Overall, PHS staff perceived the program as relevant for this target population. However, they experienced the organization and execution of strategies as time-consuming. All strategies required a flexible attitude from TB care staff to organize promotion activities — and some LTBI screenings — on location or outside office hours. Most PHS staff doubted feasibility to execute the activities in regular practice with current available resources. Furthermore, PHS staff questioned the effectiveness of the program because of low LTBI screening uptake.

#### Program facilitators

Between the different strategies, we identified the following overarching facilitators: 1) active, face-to-face outreach to the community, and 2) engagement of key figures. Furthermore, respondents suggested that repeating the information and screening opportunities would increase uptake of the program.

*Eritrean respondent: “People keep saying they are healthy, but we all said the same thing. I never had any complaints; I was not coughing. Still it was sleeping in my body. Now we can prevent it from developing into the TB disease. Therefore, we should share our experience with those who didn’t come, if you could organize a health education seminar again.” [Individual interview 7]*

*Key figure 2: “They need time to really understand the purpose and importance. ( … ) So, several announcements and several registration opportunities. After the first time, they will share their experience [with LTBI screening] among each other. Then organize a second time. Eventually, it will gain publicity and then they will cooperate.” [Group interview PHS 4]*

##### Active face-to-face outreach to the community

Strategies that actively approached smaller groups in a face-to-face manner (Strategies 2, 4, 5) had highest uptake of LTBI education and screening. Key figures explained that face-to-face explanation is effective as it allows them to explain and emphasize the importance of the program, and immediately address misunderstandings or scepticism. Contrary strategies (strategies 1, 3.3 and 6.2) used written materials such as letters, flyers and posters. Respondents described those strategies as less effective because of the overload of information from different organizations that is sent to Eritreans who recently migrated (61% of those screened for LTBI, migrated less than 3 years ago). Many Eritreans have difficulties understanding and prioritizing invitations. Consequently, they only take letters from the municipality into consideration, which can be recognized by their envelope and are known to contain compulsory appointments.

##### Engagement of key figures and community members

Most PHS staff said that the key figures were crucial in approaching and reaching the target population. Key figures from PHS 3 were very well connected to the community: they were young, from the same generation of migrants, and thus their acquaintance already originated during the journey to the Netherlands. However, respondents from PHS 1 and 2 reported mistrust and lack of respect towards key figures. Eritrean key figures — often also functioning as interpreters — who migrated during the nineties are often perceived as supporters of the current Eritrean regime, from which the new generation of Eritrean migrants fled. Mistrust towards those key figures is further perpetuated by media and public discourse of incidents where Dutch immigration authorities have expelled interpreters because of their connection to the Eritrean government.

*Key figure: “The young generation do not trust the key figures who have been in the Netherlands for 20 years. They [young generation] think that certain things happen to them personally because of the key figures, because they are the translators and are always around procedures such as housing.” [Group interview PHS 1]*

To overcome the issue of mistrust and to make future campaigns more appealing, some interview respondents suggested to engage Eritreans from the same generation who have participated in the program, for example through short promotion films.

#### Progra1m barriers

We identified the following overarching barriers: 1) competing priorities, 2) perceived good health and poor risk perception, and 3) scepticism about the project’s purpose***.*** Additional file 5 provides an overview of strategy specific facilitators, barriers and suggestions for future improvements [see Additional file 5].

##### Competing priorities of the target population

Key figures said that it was sometimes difficult to motivate the target population to participate in the program because of competing priorities (Strategy 3.1 and 4.2). Some community members are occupied with pressing issues such as housing, family reunification, Dutch language school appointments and examinations, and employment, hence influencing participation in the program.

*TB nurse: “The men said: ‘I thought you guys came to tell us something about TB related to our housing condition. If not, why would I come? I don’t care if I have TB, anything better than living in this house’.” [Group interview PHS 2]*

##### Perceived good health and poor risk perception

Some key figures said that at first the target population did not understand the relevance of attending the education about TB because they felt healthy, had a normal chest X-ray for TB at entry, and were unfamiliar with LTBI. Furthermore, the young age of some participants (Strategy 3.1) influenced the ability to relate the information to one’s own health: despite the education they felt the disease would not affect them.

*Eritrean respondent: “I participated only because I was at home. If I had a trip somewhere, I would not have come. I always thought I was healthy, and the education was not important. ( … ) I only found out that I had LTBI because I did the blood test. So, I learned a lesson from my situation, and I try to explain it to others.” [Individual interview 6]*

##### Stigma and scepticism about the project’s purpose

Some respondents felt stigmatized by the fact that the program targeted only Eritreans and not Arabic migrants. It made them feel like only Eritreans “*brought TB to the Netherlands”*. Furthermore, some respondents were sceptical about the project’s purpose. They suspected the “real” project’s goal was to test a new diagnostic test for TB. One key figure explained that this scepticism comes from gossip in the community about Western countries testing medical devices on African refugees, such as vaccines. Despite addressing these concerns, the scepticism may have resulted in negative peer pressure to participate in LTBI education and screening, especially in Strategy 6.2.

*Eritrean participant: “They said this is a pilot project to do a blood test for TB. What do you say about the fact that they are testing it on us? They did not test it on the Arab people? What if the virus stays in the needle, they are using to test this new method and it infects us? It is normal to be sceptical about this.” [Group interview 3]*

## Discussion

We engaged Eritrean key figures and TB care staff in developing and evaluating culturally appropriate strategies to reach and motivate Eritrean communities to participate in an LTBI screening and treatment program. Strategies in which key figures were well connected to the community, applied face-to-face promotion with individuals or small groups of community members, and in which participants brought friends and family to the LTBI screening were most successful in terms of LTBI education and uptake. After the education session, most participants perceived the LTBI screening as important and appreciated the opportunity to get educated, tested -and if necessary- treated, free-of-charge. Twelve percent of the screened population had LTBI. LTBI treatment initiation and completion proportions were very high (both 97%).

In line with other study findings, we could reach the target group in places like churches, football clubs, language classes and community centres [10, 25, 26]. However, uptake of LTBI screening was suboptimal. Whereas Walker et al. showed great success, reaching LTBI screening uptake of 75%, by using English language classes as outreach activities, targeting communities through Dutch language classes was one of the least successful strategies in our study [27]. In Walkers’ project the ownership and shared responsibility among school management and staff -which was limited in our study- was likely key to their success. Furthermore, the LTBI screening uptake in our study is also low compared to the uptake of contact investigation (LTBI screening) among foreign-born individuals in the Netherlands (77%) [28]. The difference could be, partly, explained by the lack of intrinsic motivation among our target group: they are no recent (close) contacts of TB patients and therefore do not feel at high risk. Other barriers for program uptake were: other pressing priorities of participants, perceived stigma and scepticism towards the project, and perceived good health and misconceptions about TB susceptibility. These are in line with previous study findings [9, 11, 29]. The latter two barriers can be addressed through health and TB education, however, this may not address the challenge to motivate individuals to attend the education session.

To overcome barriers for uptake of the program, studies have identified the engagement of community members and stakeholders in the design and execution of the project as highly valuable [5, 10–15, 25]. Some of the key figures engaged in our study were from a different migrant generation and were mistrusted by the Eritrean participants in our study. This may have impeded the uptake of the program and shows that a culture sensitive approach includes attention for cultural, political and religious differences within populations and even communities.

We tend to measure the success of an LTBI education and screening program solely by LTBI screening uptake. However, LTBI treatment initiation and completion proportions in this program were higher than those observed among TB contacts in the Netherlands [28]. This shows that the culturally appropriate approach of the program is highly successful in motivating high risk Eritrean migrants to accept and complete LTBI treatment. Additionally, our study shows that encouragement of educated community members to motivate and bring family and friends to the LTBI screening can lead to additional participants attending the LTBI screening. Furthermore, creating awareness among community members through education could decrease stigma and potential future diagnostic delays [9] and improve uptake of future LTBI education and screening activities [10].

This study only targeted Eritreans because they currently have the highest TB incidence among migrants living in the Netherlands [1, 2]. We are therefore limited in extrapolating study results to other migrant populations. However, considering the overlap of the study’s identified barriers with those identified in previous literature, one might expect to find similar barriers when working with other migrant populations. We offered LTBI treatment free of charge, whereas normally the cost of medication is deducted from the obligatory deductible excess (385 euros) for health insurance in the Netherlands. We therefore do not know what effect out of pocket expenditure for treatment would have on LTBI treatment acceptance. Despite these limitations, this study is unique in its evaluation and comparison of multiple strategies to reach and motivate a target population for TB prevention programs. Our study therefore provides evidence-based information on the use of strategies which before were used more naturally and intuitively.

PHS staff considered the program as time demanding and requiring extended organizational flexibility. Given the unpredictable uptake of the screening, they doubted the feasibility and effectiveness of the program in daily practice. Yet, the organization of TB contact investigation requires similar flexibility and effort, and Dutch PHSs have proven to be very effective and ingenious in the execution of TB contact investigation [28, 30]. To further inform policy-makers on the implementation of future TB prevention strategies, we are currently modelling the cost-effectiveness of migrant LTBI screening programs and its impact on TB incidence and transmission. Program effectiveness and cost effectiveness could be increased by joining forces with other (infectious) disease screening programs with mutual target populations [10, 31]. This collaboration could diminish stigma surrounding one or two particular disease(s) and meet the migrants’ unmet health needs to be tested for other (infectious) diseases [7, 10].

## Conclusion

Eritrean migrants eligible for TB prevention can be reached and motivated by engaging community members well connected and trusted by the community, using strategies which apply face-to-face promotion activities. Despite awareness-raising and culturally appropriate education, the uptake of the education session varied between PHSs and strategies and was often disappointing. Competing priorities and poor risk perception of the target population were among the main barriers for the uptake of the program. The educational program proved successful in most strategies in motivating the target population to participate in the LTBI screening and led to very high LTBI treatment initiation and completion rates among those with LTBI.

## Supplementary information

**Additional file 1.** Flowchart of LTBI screening and treatment process.

**Additional file 2.** Interview topic guide, Topic guide for group interviews with Eritrean participants and project team members and TB care staff, and for individual interviews with Eritrean participants.

**Additional file 3.** Descriptive statistics of the study population.

**Additional file 4.** Evaluation of LTBI treatment by the TB physician.

**Additional file 5.** Strategy specific facilitators and barriers identified in interviews with TB care staff, Eritrean key figures and Eritrean participants.

## Data Availability

The dataset generated and/or analysed during the current study are not publicly available due to the sensitivity of this study’s data and the privacy of our participants, but are available from the Dutch Tuberculosis Data Registration Committee (Henrieke.schimmel@rivm.nl) on reasonable request.

## Abbreviations

CXR: Chest X-ray
LTBI: Latent Tuberculosis Infection
PHS: Public Health Service
QFT-Plus: QuantiFERON-TB Gold Plus
TB: Tuberculosis
