# Supplementary material for: Strategies to reach and motivate migrant communities at high risk for TB to participate in a latent tuberculosis infection screening program: a community-engaged, mixed methods study among Eritreans
Source: BMC Public Health. 2020 Mar 12;20:315. doi: 10.1186/s12889-020-8390-9 (PMC7068882; doi:10.1186/s12889-020-8390-9)
Supplement: Supplementary file 1 — Additional file 1. Flowchart of LTBI screening and treatment process. [file 12889_2020_8390_MOESM1_ESM.pdf]

Additional file 1. Flowchart of LTBI-screening and treatment process

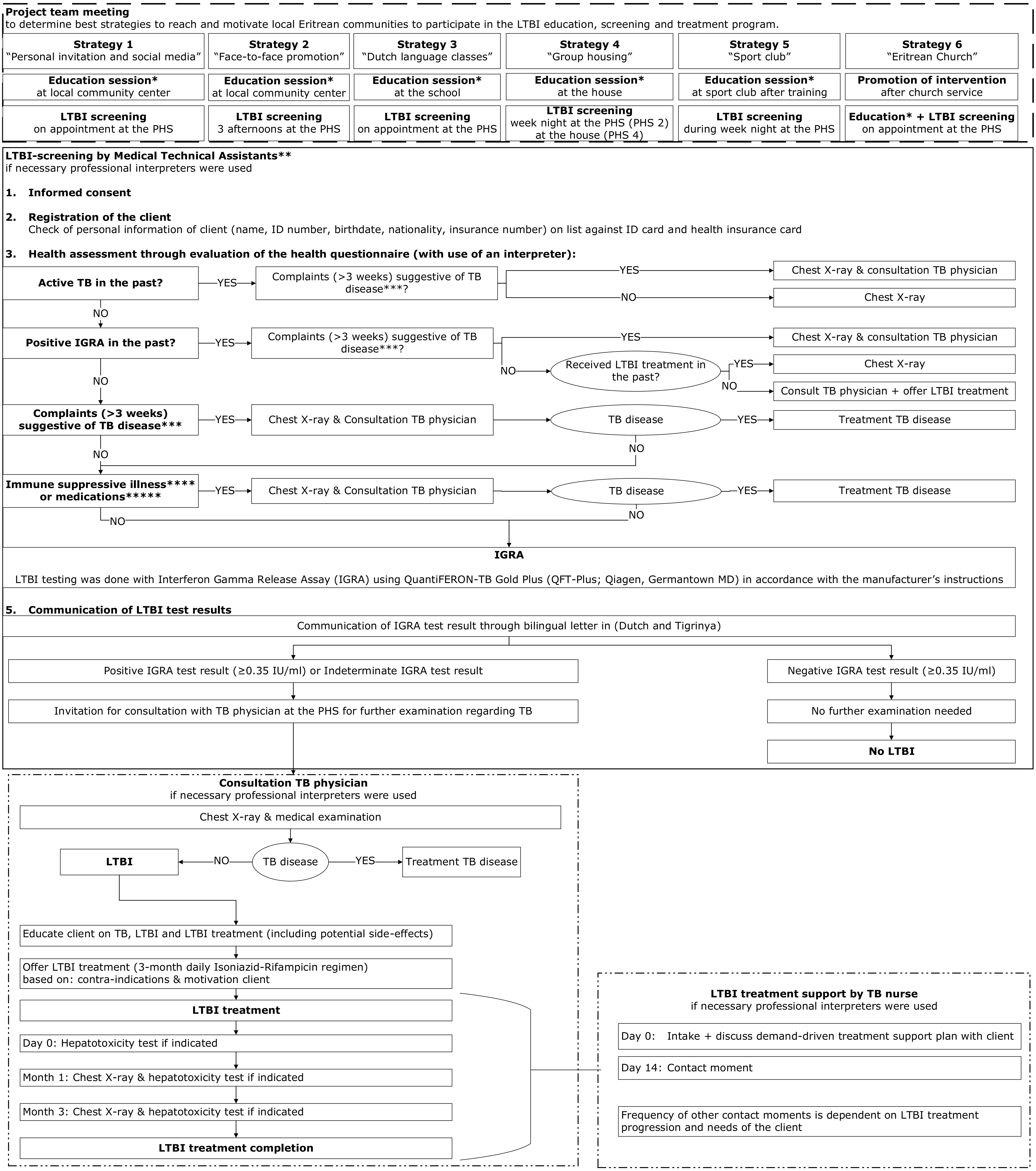

ID card identification card, *IGRA* Interferon Gamma Release Assay, *LTBI* latent tuberculosis infection, *PHS* Public Health Service, *TB* tuberculosis

*Culture appropriate education on TB, LTBI (including cause, symptoms, transmission, diagnostics and treatment) given by trained Eritrean key figures in Tirgrinya

** Medical Technical Assistants (MTA), healthcare assistants trained to perform TB screening activities (symptom screening, tuberculin skin test and chest X-ray), BCG vaccination, to give information on the scope of the screening and TB in general and to document screening results and to perform specific administrative tasks.

***Complaints: Cough, fever (>38 degrees Celsius), night sweats, poor appetite / weight loss

****Immune suppressive illnesses: Inflammatory bowel disease, kidney failure / dialysis, diabetes, HIV/AIDS, cancer, organ transplantation, psoriasis, rheumatism, sarcoidosis, silicosis

*****Immune suppressive medications: Prednisone / Dexamethasone / Methotrexate, TNF-alpha blockers (biologicals), cancer medication, medication following organ transplantation
