# Supplementary material for: Strategies to reach and motivate migrant communities at high risk for TB to participate in a latent tuberculosis infection screening program: a community-engaged, mixed methods study among Eritreans
Source: BMC Public Health. 2020 Mar 12;20:315. doi: 10.1186/s12889-020-8390-9 (PMC7068882; doi:10.1186/s12889-020-8390-9)
Supplement: Supplementary file 2 — Additional file 2. Interview topic guide, Topic guide for group interviews with Eritrean participants and project team members and TB care staff, and for individual interviews with Eritrean participants. [file 12889_2020_8390_MOESM2_ESM.pdf]

Additional File 2. Interview Topic Guide

1. Group interviews among Eritrean participants

Communication of the intervention

1. How did you hear about the education session about tuberculosis?
   Who told you about the health education about tuberculosis?
2. What do you think about the way you were approach for the health education session?
   Do you think it could have been done in a different or better way? Explain.
   Do you think another method would have been better in reaching more Eritrean migrants?
3. Can you describe your first reaction to the invitation to come to the health education about tuberculosis?
4. What did you think about the fact that the education session was especially designed for Eritrea migrants?

Health education

1. What made you decide to go to the health education about tuberculosis?
2. What did you expect from the health education about tuberculosis?
3. Do you think you have better knowledge now about tuberculosis? Explain.
4. Could you explain what the disease tuberculosis is?
   a. Could you explain how you can get the disease tuberculosis?
5. Could you explain the difference between the disease tuberculosis and latent tuberculosis infection?
6. We can always improve the health education, what do you think can be improved?
7. Did you discuss the health education with other people? What did you discuss?

LTBI screening

1. What made you decide to go to the LTBI screening?
2. What was your reaction to the possibility to get the free screening for LTBI?
3. Did you tell other people about the opportunity to get the LTBI screening? How did they react?
4. What did you expect of the LTBI screening today?
   Do you think you were well informed about what to expect today and how the screening was organized?
5. Do you know why the LTBI screening today was organized? Explain.
6. Do you know what the outcomes of the screening / blood test can be? Explain.
7. If the blood test shows you are infected with the tuberculosis bacteria, do you know what the follow-up / next steps will be?

Stigma and screening for (latent) TB

1. Do you feel comfortable talking about the tuberculosis screening with other people?
   If yes:
   a. What would you discuss?
   b. With whom?
   If no:
   c. Why not? What would help you?
2. Do you feel concerned about confidentiality in a group screening?
3. How do you think other people would react if you tell them about the LTBI screening?
   a. How do you feel about their reaction?
4. Do you feel free to cooperate with the screening?
5. Do you feel free to receive written information about TB / screening? (or rather hide this from others). Or look at the internet for information freely (also when others can see this?)

2. Individual interviews among Eritrean participants

Positive LTBI test result and initiation of preventive treatment

1. What was your reaction when you read in the letter that you had a positive blood test?
2. Did you share the result of the test with anyone?
3. The letter invited you for an appointment with the doctor. Can you explain how your first consultation with the doctor went?
   a. What did they tell you about your results of the blood test?
   b. What did they tell you about tuberculosis and LTBI?
   c. What did they tell you about the treatment?
4. Did the doctor gave you any other information material about LTBI and the treatment?
   a. For example: an information brochure?
5. How did you feel after your first consultation with the doctor?
   a. Could you remember all the information that you were told?
6. How did you feel when you were about to start the treatment?
   a. Did you feel confident?
7. When did you start your treatment?
8. You had to get your medication from the pharmacy. How did that go?

Consultation TB care staff

1. Did you discuss with your TB nurse how they could best assist you during your treatment? Explain.
2. Can you tell us about the support that the nurses and the doctor gave during the preventive treatment?
   b. What do you think of the support that you received?

    c. Did you have any saying in the type of support that you got? Was the support during the treatment mutually discussed?
3. How often have you been in contact with the nurse / doctor?
    a. How did you have contact? Telephone / visit MHS / house visit by nurse?
    b. What did you think about this contact?
    c. Did you understand what was told during the contact with the nurse / doctor?
    d. Was there enough time to discuss your questions with the doctor / nurse?
4. How did you travel to the MHS?
    a. How often did you have to travel to the MHS?
    b. Was the MHS easy to find the first time you visited the MHS?
    c. Was it easy to travel to the MHS (regarding time and transitions (bus, train etc.))?
    d. Did you have to pay the travel costs or any other costs yourself?

Support during treatment

1. Did you tell anyone about your preventive treatment?
    d. Who? How did they react? Were they supportive? How?
2. Did you receive any support from other people than the MHS staff during your treatment?
    If yes:
    a. From whom? What kind of support? Did it help you?
    If not:
    b. Would you have wished that there was other or extra support?
    c. What kind of support?

Stigma

1. In your community, how is a person who has LTBI usually regarded/treated?
2. Did you feel comfortable to discuss your LTBI and preventive treatment with other people in your community?
3. How did people react to the fact that you took preventive treatment for tuberculosis infection? / How do you think people would have reacted (if you told them)?

Communication

1. How did you communicate with your doctor / nurse?
2. How did you experience the interaction with the doctor / nurse? Explain.
3. What did you think about communicating using an interpreter?
4. Did you feel comfortable asking questions to you doctor / nurse? Explain.

Treatment compliance

1. Can you explain why you started treatment for latent tuberculosis infection?
   Can you explain why you did not start treatment for latent tuberculosis infection?

2. Can you tell me something about the duration of the treatment and the amount of pills?
    b. What do you think about the duration of the treatment?
    c. Why takes the treatment 3 months?
3. Did the TB nurse give you any advice or tips on how to remember to take your medication?
    a. What advice / tips did you receive?
    b. Did it help you?
    c. Did you come up with tricks yourself to remind yourself to take your medication?
4. Can you explain why it is important to take the pills every day?
5. Why was it so important for you to finish your treatment?
6. Did you find it difficult to take the pills every day?
    a. What moments were difficult? Why?
7. Did you sometimes forget to take your pills?
    a. What did you do when you forgot to take your pills?
    b. Can you tell me how you felt when you forgot to take your pills?
    c. Can you tell me how many times you have forgotten your pills?
    d. Have you talked about it with your doctor or your nurse?
8. Were there moments when you wanted to quit the preventive treatment?

    If no:

    a. What kept you going?

    If yes:

    b. Why did you wanted to quit the treatment?

    c. What did you do when you wanted to quit the preventive treatment?

Side effects

1. Did you receive information about potential side effects of the treatment? Explain.
2. Did you experience any side effects from the treatment? Explain.

3. Group interviews among project team members and tuberculosis care staff

Preparation work

1. Can you (Public Health study coordinator) explain -step by step- how you recruited members for the project team?
2. Can you explain -step by step- how you identified the community in your region?
3. Can you explain -step by step- how and which strategies you developed and planned to reach and motivate Eritrean community members?
    a. Which challenges did you face? What did you do to overcome these challenges?
4. Can you explain -step by step- how you executed the strategies?
    a. Which challenges did you face?

b. Which factors facilitated the execution of the strategies?
c. Which factors impeded the execution of the strategies? How did you deal with barriers?

TB and LTBI Education session

1. What is your opinion about the content and value of the TB/LTBI education session?
2. How do you think participants received the TB/LTBI education?
3. Can you describe how the LTBI/TB education was organized?
4. Can you describe the preparations that were taken to organize the TB/LTBI education?
5. What would you do different in organizing and executing the TB/LTBI education in the future?
6. What would you do similar in organizing and executing the TB/LTBI education in the future?

LTBI screening

7. Can you describe how the LTBI screening was organized?
8. Can you describe the preparations that were taken to organize the LTBI screening?
9. What would you do different in organizing the LTBI screening in the future?
10. What would you do similar in organizing the LTBI screening in the future?
11. How did you experience the LTBI screening?
12. What do you think about the participation (show up rates) of the LTBI screening?
    a. What did you do in advance to enhance the participation rates?
    b. How do you think you can improve the participation of the LTBI screening in the future?
13. Did you encounter language barriers?
    a. How did you address these language barriers?
14. Can you describe how you handled the health questionnaire?
    a. How did the participants answer the health questionnaire?
    b. Did you discuss the answers with the participants? How?
    c. Did you encounter any barriers collecting information through the health questionnaire?
       If yes:
       i. How would you overcome the barriers in the future?
    d. Do you think the HQ is useful?
15. Can you describe how the blood collection for the IGRA was performed?
    a. Did you experience or notice any difficulties during the collection of blood?
       If yes:
       i. How would you overcome these difficulties in the future?
    b. Can you describe how blood is further transported to the lab?
16. Have you observed and can you describe cultural factors that influenced the participation of the LTBI screening?
    a. How did you cope with these cultural factors?

17. What would you change in future LTBI screening events targeting high-TB-risk migrant communities?

Preventive treatment

18. What do you think about the usefulness and effectiveness of the LTBI screening of high-TB-risk migrant populations such as the Eritreans?
19. Could you (TB physician) describe how the first consultation with LTBI clients went?
    a. How did people react to the fact that there were infected with tuberculosis?
    b. What do you tell / talk about in the first consultation?
    c. How do you offer preventive treatment?
        i. How do you direct a conversation, for example if you really want someone to start preventive treatment / if you don't think the preventive treatment is going to be a success?
        ii. Which factors do you consider when offering preventive treatment / pushing for X-ray?
    d. What kind of barriers did you encounter?
    e. How did you overcome these barriers?
20. What factors do you think contributed to the acceptance adherence/continuation/completion of the preventive treatment?
21. What factors do you think impeded the acceptance, adherence/continuation/completion of the preventive treatment?
    a. How would you overcome these barriers?
22. Can you describe how the support of patients with preventive treatment is organized?
23. What factors in patient support do you think contributed to the continuing and completion of the preventive treatment?
24. Did you use the material for the "health education" provided from the project? Was it useful and appropriate? What unmet needs do you have regarding the "health education" material?
25. What cultural factors did you encounter during the patient support for the preventive treatment? Do you think these factors influenced treatment acceptance and adherence? How did you cope with impeding cultural factors?
26. Can you describe how the communication took place with the client?
    a. How did you cope with difficulties in communication?
    b. How often did you make use of an interpreter?
    c. What are advantages and disadvantages of using interpreter?
27. If you could give recommendations to another MHS office, who has no experience yet with the LTBI screening and preventive treatment, what would you advise them?
