# Supplementary material for: Strategies to reach and motivate migrant communities at high risk for TB to participate in a latent tuberculosis infection screening program: a community-engaged, mixed methods study among Eritreans
Source: BMC Public Health. 2020 Mar 12;20:315. doi: 10.1186/s12889-020-8390-9 (PMC7068882; doi:10.1186/s12889-020-8390-9)
Supplement: Supplementary file 3 — Additional file 3. Descriptive statistics of the study population. [file 12889_2020_8390_MOESM3_ESM.pdf]

Additional file 2. Descriptive statistics of the study population

	**LTBI screening**		**LTBI diagnosis**		**Initiated LTBI treatment**		**Completed LTBI treatment**	
**Characteristic**	**n**	**Column %**	**n**	**Row %**	**n**	**Row %**	**n**	**Row %**
**Total**	257	(100%)	30	(12%)	29	(97%)	28	(97%)
**Public Health Service (PHS)**								
PHS 1	35	(14%)	2	(6%)	2	(100%)	1	(50%)
PHS 2	48	(19%)	8	(17%)	7	(88%)	7	(100%)
PHS 3	62	(24%)	12	(19%)	12	(100%)	12	(100%)
PHS 4	101	(28%)	8	(8%)	8	(100%)	8	(100%)
PHS 5	10	(3%)	0	(0%)	-	-		-
**Gender**								
Female	129	(50%)	10	(8%)	10	(100%)	10	(100%)
Male	128	(50%)	20	(16%)	19	(95%)	18	(95%)
**Age category**								
0-17 years	48	(19%)	1	(2%)	1	(100%)	1	(100%)
18-24 years	63	(25%)	5	(8%)	5	(100%)	4	(80%)
25-34 years	103	(40%)	12	(12%)	12	(100%)	12	(100%)
35-44 years	43	(17%)	12	(28%)	11	(92%)	11	(100%)
**Household composition**								
Alone	122	(45%)	16	(13%)	16	(100%)	15	(94%)
With family	123	(48%)	11	(9%)	10	(91%)	10	(100%)
Missing	12	(5%)	3	(25%)	3	(100%)	3	(100%)
**Education level**								
No formal / primary education	116	(45%)	12	(10%)	11	(92%)	11	(100%)
Secondary school	108	(42%)	13	(12%)	13	(100%)	12	(92%)
Higher education	28	(11%)	2	(7%)	2	(100%)	2	(100%)
Missing	5	(2%)	3	(60%)	3	(100%)	3	(100%)
**Employment status**								
Employed (payed job)	4	(2%)	0	(0%)	-	-	-	-
Unemployed (no payed job)	35	(14%)	6	(17%)	5	(83%)	5	(100%)
Student*	210	(82%)	22	(10%)	22	(100%)	21	(95%)
Missing	8	(3%)	2	(25%)	2	(100%)	2	(100%)
**Duration of stay in the Netherlands**								
<6 months	12	(5%)	1		0	(0%)		
≥6 months, <1 year	31	(12%)	2		2	(100%)	2	(93%)
≥1 year, <2 years	25	(10%)	1		1	(100%)	1	(100%)
≥2 years, <3 years	89	(35%)	14		14	(100%)	13	(93%)
≥3 years, <10 years	11	(4%)	4		4	(100%)	4	(100%)
Missing	90	(35%)	8		8	(100%)	8	(100%)

LTBI latent tuberculosis infection, *PHS* public health service, *TB* tuberculosis

*Includes: primary school, secondary school, college, integration courses
