# Supplementary material for: Strategies to reach and motivate migrant communities at high risk for TB to participate in a latent tuberculosis infection screening program: a community-engaged, mixed methods study among Eritreans
Source: BMC Public Health. 2020 Mar 12;20:315. doi: 10.1186/s12889-020-8390-9 (PMC7068882; doi:10.1186/s12889-020-8390-9)
Supplement: Supplementary file 4 — Additional file 4. Evaluation of LTBI treatment by the TB physician. [file 12889_2020_8390_MOESM4_ESM.pdf]

Additional file 3. Evaluation of LTBI treatment by the TB physician

	n	(%)
**Total clients diagnosed with LTBI**	30	(12%)
**Was a language a barrier and was an interpreter used during consultations?**		
No, it was no barrier and no interpreter was used	0	-
Yes, it was a little a barrier, but no interpreter was needed	2	(7%)
Yes, it was a little a barrier, but no interpreter was available	0	-
Yes, it was a barrier and a professional interpreter was used	13	(45%)
Yes, it was a barrier and the TB nurse interpreted and supported the client	14	(48%)
**Initiation of LTBI treatment**		
Yes	29	(97%)
No, because of low IGRA value	1	(3%)
**Reported side-effects during LTBI treatment**		
Yes[1]	7	(24%)
No	20	(69%)
Missing	2	(7%)
**Challenges reported by PHS staff during LTBI treatment**		
Yes	8	(28%)
No	18	(62%)
Missing	3	(10%)
**Type of challenge reported during LTBI treatment**		
Compliance / Adherence	1	(3%)
Difficulties follow-up appointments	1	(3%)
Difficulties duration of PT / amount of pills	1	(3%)
Afraid of blood sampling	1	(3%)
Difficult to not drink alcohol	1	(3%)
Difficult to reach by phone	1	(3%)
Difficulties understanding LTBI	1	(3%)
**LTBI treatment support given by TB nurse**		
Yes	29	(100%)
< 10 times	27	(93%)
≥ 10 times	1	(3%)
Missing	1	(3%)
**Client received Directly Observed Therapy**		
No	29	(100%)
**LTBI treatment result**		
Completed[2]	28	(97%)

LTBI Latent tuberculosis infection, *PHS* Public Health Service, *TB* Tuberculosis

[1] Side effects reported: hepatotoxicity (n=3)

[2] LTBI treatment discontinued (n=1) due to side-effects (psychosis)
