# Supplementary material for: Strategies to reach and motivate migrant communities at high risk for TB to participate in a latent tuberculosis infection screening program: a community-engaged, mixed methods study among Eritreans
Source: BMC Public Health. 2020 Mar 12;20:315. doi: 10.1186/s12889-020-8390-9 (PMC7068882; doi:10.1186/s12889-020-8390-9)
Supplement: Supplementary file 5 — Additional file 5. Strategy specific facilitators and barriers identified in interviews with TB care staff, Eritrean key figures and Eritrean participants. [file 12889_2020_8390_MOESM5_ESM.pdf]

Additional file 4. Strategy specific facilitators and barriers identified in interviews with TB care staff, Eritrean key figures and Eritrean participants

**Strategy 1: Invitation through mail and social media**	
Barriers	- Shortcoming project team: no coherence between invitations presented in mail and social media (PHS 1)<br>- Target group receives invitations from various organizations, including from other departments of the PHS. The key figures and participants said they were overwhelmed by these invitations and have difficulty prioritizing the relevance and importance of the invitations. (PHS 1)<br>- Clashing appointments for LTBI-screening with school or other obligations (PHS 1)
Facilitators	- Education session was organized during week night to avoid clashing appointments (PHS 1)<br>- Some key figures -those asked to mobilize their network- were not trusted by the community as they were from a different generation of Eritrean migrants (PHS 1)
Suggestions	- Send invitation through municipality, this has a compulsory character (PHS 1)<br>- Written invitations should put more emphasis on importance and relevance of the education session and screening. (PHS 1)<br>- Community-members from the same age-group and generation of migrants should be appointed as key figures to promote the program (PHS 1)
**Strategy 2: Face-to-face promotion**	
Barriers	- Some participants felt stigmatized because the program only targeted Eritreans, and no other nationalities. (PHS 3)
Facilitators	- Active encouragement by PHS staff to bring friends and family to the screening (PHS 3)<br>- PHS staff organized an extra screening opportunity during which approximately 15 additional persons showed up. (PHS 3)
Suggestions	- Repeat the program: now that it has gained trust and familiarity within the Eritrean community, a second round would likely reach more people.<br>- Involve persons treated for LTBI to share their experience and promote the program.
**Strategy 3: Dutch language classes**	
Barriers	- Participants felt uncomfortable about being visibly separated for the education session at school from school/classmates with Dutch or other nationalities (PHS 1)<br>- Education had to be organized during break hours. However, the students value their break hours and rather relax with each other (PHS 1)<br>- Young age of group, who perceived themselves as healthy and strong and therefore think it is very unlikely they will be affected by TB (PHS 1)

	- Lack of respect for key figures by some participants: perceived condescending attitude of key figures towards participants (PHS 2)<br>- Dependency of PHS on willingness and efforts made of school staff to approach and motivate students (PHS 2)<br>- Passive approach – displaying posters and handing-out flyers without further promotional talk (PHS 2)
Facilitators	- Their presence was assured since they are present at school for their regular classes (PHS 1)<br>- Educational setting which is suitable for health education (PHS 1)<br>- Active and face-to-face approach to motivate students by the key figures (PHS 2)
Suggestions	- Involve school management and teachers more intensive and early on in the organization of the strategy and ensure a feeling of shared responsibility to make the strategy a success
**Strategy 4: Group house**	
Barriers	- Participants had a lack of understanding of importance of LTBI education and screening because previous chest X-rays were all normal. So, no need for further education and testing (PHS 2)<br>- Participants had other priorities, such as moving to an individual place to live (PHS 2)<br>- Participants were fed-up with all the organizations approaching them, without addressing their needs (moving to an individual house). (PHS 2)<br>- Negative peer pressure among roommates to not attend the education session (PHS 2)<br>- Participants perceived themselves as healthy and thus no need for education (PHS 4)
Facilitators	- Target group resides in one place, which facilitated easy access. (PHS 2 and 4)<br>- Education can be organized at their residence, so that there is no need to travel (PHS 2)<br>- Target group resides in one place, which facilitated easy access. (PHS 2)<br>- Education can be organized at their residence: there is no need to travel (PHS 2)<br>- After education they perceived the screening as important as motivated and mobilized friends (PHS 4)<br>- Positive peer pressure from roommates to participate in education session and LTBI screening. (PHS 4)<br>- In consultation with residents, LTBI screening was organized at the house: there was no need to travel (PHS 4)
Suggestions	- Organize LTBI screening at the house, so there is no need for the group of participants to travel.<br>- Stimulate participants to bring roommates (who did not attend education session), friends and family members to the LTBI screening
**Strategy 5: Male football team**	
Barriers	- None
Facilitators	- Ensured presence at the location of the education session because of football training (PHS 2)<br>- Education session was promoted by the -respected- football coach (PHS 2)<br>- Positive peer pressure on deciding to participate: some of them showed open enthusiasm (PHS 2)
Suggestions	- None

**Strategy 6: Eritrean church**	
Barriers	- Persons are tired and hungry after a 3 to 4-hour church service before and during which no food is allowed. This may influence the ability of persons to absorb new information (PHS 4)<br>- Participants may have forgotten about the screening because of two-week time gap, including Easter celebration. (PHS 5)<br>- Skepticism about purpose of the project: suspicion about being used for testing a diagnostic test (PHS 5)<br>- Participants felt stigmatized because the program only targeted Eritreans, and no other nationalities (PHS 5)<br>- The church is a safe and holy place for Eritrean church members. For this reason, they may have perceived the brief education session and promotion of LTBI screening at the church as disrespectful and intruding. (PHS 5)
Facilitators	- Support for the program by the priest is likely to motivate church members to participate (PHS 4)
Suggestions	- The key figures said it is likely that church members may have forgotten about the information given during the brief education session because church members are tired after a 3 to 4-hour service without food. Organize the announcement and promotion more than once, as well as multiple screening opportunities so that church members remember information and can share experiences among each other (PHS 4)<br>- Announcement preferably by the priest (or other much respected church member themselves) (PHS 5)

LTBI Latent tuberculosis infection, *PHS* Public Health Service, *TB* Tuberculosis
